# The Superiority of Removable Contact Splints in the Healing of Diabetic Foot during Postoperative Care

**DOI:** 10.1155/2019/5945839

**Published:** 2019-09-15

**Authors:** Vladimíra Fejfarová, Jaroslav Pavlů, Robert Bém, Veronika Wosková, Michal Dubský, Andrea Němcová, Alexandra Jirkovská, Bedřich Sixta, Karol Sutoris, Filip Thieme, David G. Armstrong, Eliška Vrátná, Jitka Hazdrová, Věra Lánská

**Affiliations:** ^1^Diabetes Centre, Institute for Clinical and Experimental Medicine, Prague, Czech Republic; ^2^Department of Transplant Surgery, Institute for Clinical and Experimental Medicine, Prague, Czech Republic; ^3^Southwestern Academic Limb Salvage Alliance (SALSA), University of Southern California (USC), Los Angeles, USA

## Abstract

**Objective:**

Off-loading is one of the crucial components of diabetic foot (DF) therapy. However, there remains a paucity of studies on the most suitable off-loading for DF patients under postoperative care. The aim of our study was to evaluate the effect of different protective off-loading devices on healing and postoperative complications in DF patients following limb preservation surgery.

**Methods:**

This observational study comprised 127 DF patients. All enrolled patients had undergone foot surgery and were off-loaded empirically as follows: wheelchair+removable contact splint (RCS) (group R: 29.2%), wheelchair only (group W: 48%), and wheelchair+removable prefabricated device (group WP: 22.8%). We compared the primary (e.g., the number of healed patients, healing time, and duration of antibiotic (ATB) therapy) and secondary outcomes (e.g., number of reamputations and number and duration of rehospitalizations) with regard to the operation regions across all study groups.

**Results:**

The lowest number of postoperative complications (number of reamputations: *p* = 0.028; rehospitalizations: *p* = 0.0085; and major amputations: *p* = 0.02) was in group R compared to groups W and WP. There was a strong trend toward a higher percentage of healed patients (78.4% vs. 55.7% and 65.5%; *p* = 0.068) over a shorter duration (13.7 vs. 16.5 and 20.3 weeks; *p* = 0.055) in the R group, as well. Furthermore, our subanalysis revealed better primary outcomes in patients operated in the midfoot and better secondary outcomes in patients after forefoot surgery—odds ratios favouring the R group included healing at 2.5 (95% CI, 1.04-6.15; *p* = 0.037), reamputations at 0.32 (95% CI, 0.12-0.84; *p* = 0.018), and rehospitalizations at 0.22 (95% CI, 0.08-0.58; *p* = 0.0013).

**Conclusions:**

This observational study suggests that removable contact splint combined with a wheelchair is better than a wheelchair with or without removable off-loading device for accelerating wound healing after surgical procedures; it also minimises overall postoperative complications, reducing the number of reamputations by up to 77% and the number of rehospitalizations by up to 66%.

## 1. Introduction

One of the most important components of diabetic foot (DF) therapy is appropriate off-loading [1–4]. DF off-loading improves healing by immobilising and reducing maximal plantar pressure [5]. Several therapeutic devices are routinely used to treat DF patients. The total contact cast (TCC) is the most efficient and considered the gold standard for DF off-loading [6, 7]. Other off-loading options are half-shoes and prefabricated or individualized orthoses [1, 8–10]. These tools are easier to prescribe, but they do not always result inefficient healing [10–12]. A wheelchair is usually recommended in cases of extensive foot lesions and bilateral impairment.

Many studies describe the effectiveness of different off-loading modalities, especially the TCC in the treatment of DF ulcers or Charcot osteoarthropathy [6]. However, none of these studies has clearly proved which type of off-loading should be used for DF patients under postoperative care. We have experience with TCCs in the treatment of DF ulcers of varying degrees [13], but most do not use them for postoperative care. To improve access to the surgical wound and facilitate faster and easier availability, we modified the TCC application method by using removable contact splints (RCSs) of various types (L-dorsal, L-pretibial, and U-splint) to ensure adequate stability and immobilisation. The aims of our study were to evaluate the effectiveness of various off-loading methods used for DF patients who had undergone extensive foot surgery at different intervention locations and to confirm the effectiveness of our novel off-loading method—removable contact splints (RCSs)—toward achieving better postoperative outcomes.

## 2. Research Design and Methods

### 2.1. Study Subjects

Annually, approximately 130 to 200 patients undergo a surgical procedure in our centre—only 50% of them were hospitalized patients. We included in our study about 20% of our clients treated only by one type of off-loading device, since the type of off-loading method is frequently changed in the majority of patients, which could negatively affect the evaluation of the effect of certain off-loading device. Based on our inclusion criteria, our prospective observational comparative study comprised 127 patients treated for DF (DF ulcers scored by the University of Texas (UT) diabetic wound classification stages as 2B/3B or 2D/3D; [14]) at the foot clinic of the Diabetes Centre at the Institute for Clinical and Experimental Medicine (from 06/2013 to 06/2017). All patients who have undergone at least one surgical foot procedure were off-loaded using one type of off-loading method only during the whole observed period. Patients were followed until they were healed or for at least 3 months up to a maximum of 12 months (26.3 weeks on average). Exclusion criteria included postsurgical follow-up shorter than 3 months, use of more than one off-loading method during the follow-up period, full nonadherence to off-loading (defined as wearing of off-loading less than 50% of the time during the day as we usually recommended), and complete immobility due to stroke, paresis, or other neurological disabilities.

Patients were treated comprehensively according to the IWGDF guidance on the prevention and management of foot problems in diabetes, which seeks to improve diabetes control and the detection and therapy of macrovascular complications such as peripheral arterial disease (PAD) and infection complications [1]. In our study, off-loading was indicated empirically according to the type and extent of the surgical procedure, anatomical proportions (lower limb proportions, swelling, and the presence of deformities), and the health status of the patient, including locomotor skills. Based on the type of off-loading, patients were divided into 3 treatment groups: patients treated by RCS (our innovative off-loading method introduced to the off-loading therapy at the beginning of 2013) plus wheelchair (group R; *n* = 37; [13]); patients treated by wheelchair only (group W, *n* = 61); and those treated by a combination of a wheelchair plus a removable device (half-shoe/orthosis) (group WP; *n* = 29). Basic characteristics of the DF patients (except mean age) did not differ significantly across the study groups (Table 1). Group W differed significantly with regard to the incidence of peripheral artery disease (PAD; *p* = 0.019). However, transcutaneous oxygen pressure (TcPO_2_) values evaluated preoperation did not differ significantly among the study groups.

The primary outcomes of DF postoperative therapy—percentage, duration and costs of hospitalizations, percentage of healed patients, healing time, length of antibiotic (ATB) therapy, and number of revascularisations—were compared among the study groups. From the secondary outcomes, we assessed postoperative complications, such as the number of reamputations and major amputations and the number and duration of rehospitalizations. All outcomes were analysed over the course of the follow-up period. Results were also compared with regard to the locations of the surgical procedures performed.

Prior to enrolment into the study, each patient signed an informed consent form approved by the local ethic authority.

### 2.2. Removable Contact Splints (RCSs)

**Figure 1:**
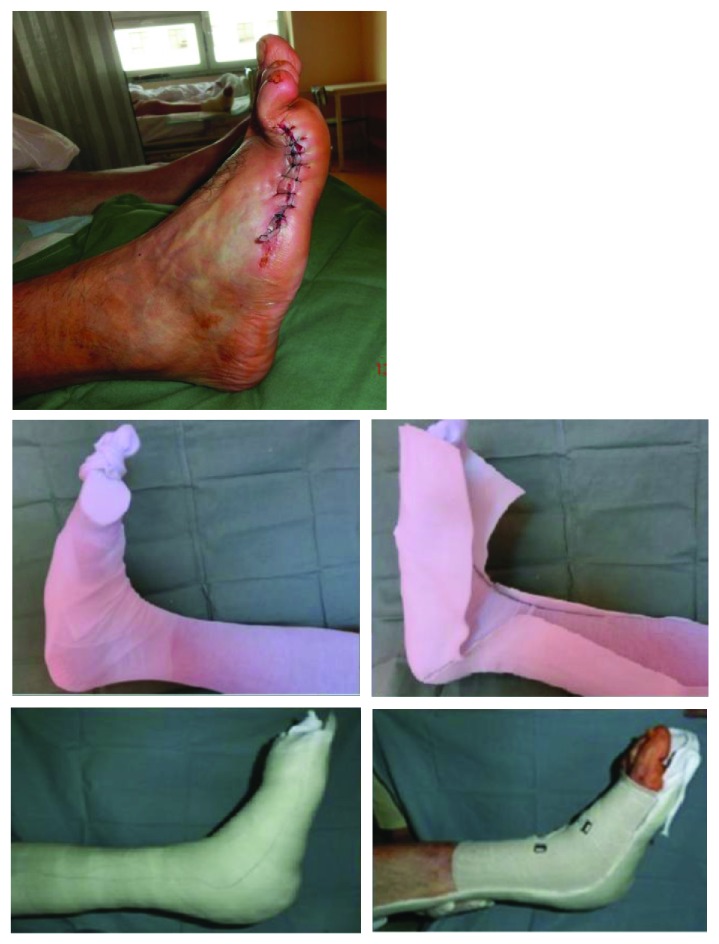
Removable contact splint manufacturing.

**Figure 2:**
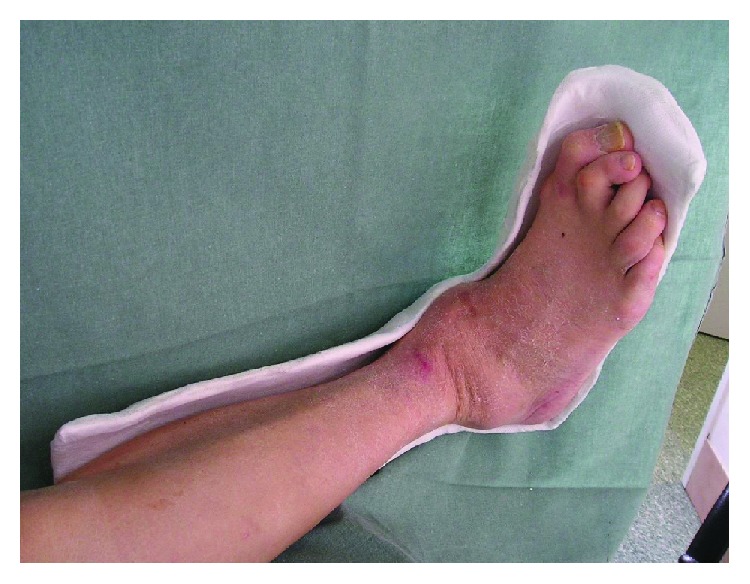
Dorsal L—removable contact splint—final product.

We developed our RCSs by modifying casting techniques. To produce the special contact splints, we used a variety of underlying materials, including stockings, cotton wool materials, adhesive felt-padding of different thickness layers, and gypsum bandages as well as semirigid and rigid bandages containing polymerising resins. The L-dorsal RCS was the most frequently produced. Manufacturing was carried out in several steps (Figure 1). We first applied a stocking along the lower limb to which we dorsally attached a thin, sticky felt material. This took the form of a splint made from a rigid material in the shape of the letter L. The material was then fixed to the lower limb using 1 or 2 rolls of semirigid bandages to make the splint compact. This product was cut along its entire length on both sides of the lower limb. The semifinished product was subsequently removed and shaped adequately to avoid compressing the toes, popliteal area, foot ulcers, or any surgical wounds. The edge was arranged so as not to partially or totally overlap bone prominences, where the risk of excoriation and foot ulcers is the greatest. The last step consisted of adapting the RCS edges by cutting and taping (Figure 2). For a more detailed description of RCS production, see the available literature [15, 16].

### 2.3. Surgical Procedures

Of the patients enrolled in the study, 61.4% (78/127) had undergone transmetatarsal toe amputations, metatarsophalangeal joint resections, transmetatarsal amputations, sesamoid bone resection, and soft tissue operations of the forefoot; 29.1% (37/127) of patients had undergone Lisfranc amputations, tarsal bone procedures, such as bone abrasions, extirpations, and resections, and soft tissue operations of the midfoot; and 9.5% of patients had undergone calcaneal resections (including abrasions) and soft tissue procedures of the hindfoot. Patients that had undergone the following procedures were excluded from the study: bone biopsies and dissections, fistula resections, ulcerectomy, simple tenolysis, Syme amputations, and major amputations.

### 2.4. Definition of Peripheral Arterial Disease

PAD was determined based on the following criteria: patient history (endovascular or surgical revascularisation), ankle brachial index (ABI) lower than 0.9 on the examined limb, and/or a TcPO_2_ value below 40 mmHg measured just before surgery. We used duplex vascular ultrasound and/or CT/MR angiography to verify vascular status.

### 2.5. Definition of Osteomyelitis

Osteomyelitis was diagnosed based on laboratory signs of inflammation, positive probe-to-bone (PTB) testing, X-ray findings, and previous osteobiopsies [1].

### 2.6. Statistical Analysis

Shapiro-Wilk's statistics was used to test the Gaussian distribution. Then, the Gaussian variables were tested by ANOVA, with the Tukey-Kramer method used for multiple comparisons. For variables differing from the Gaussian distribution, we applied the Kruskal-Wallis test and Steel-Dwass method for multiple comparisons. For discrete variables, we used *χ*^2^ test of independency in contingency tables. For statistical analysis of patient characteristics and its responsiveness and power analysis, we applied Nominal Logistic Fit test and ANCOVA test. A two-sided *p* value of less than 0.05 was considered statistically significant. The log rank test was used in Kaplan-Meier for DF healing. All calculations were carried out using JMP 11 statistical software.

## 3. Results

Approximately 14.2% of subjects were surgically treated as outpatients, while 85.8% were hospitalized. Significantly, more patients from group R were managed as inpatients (100%, *p* < 0.0001) compared to other groups (Table 1). Patients in group W had significantly longer hospital stays, leading to a significant increase in hospital costs compared to other study groups (Table 2).

**Figure 3:**
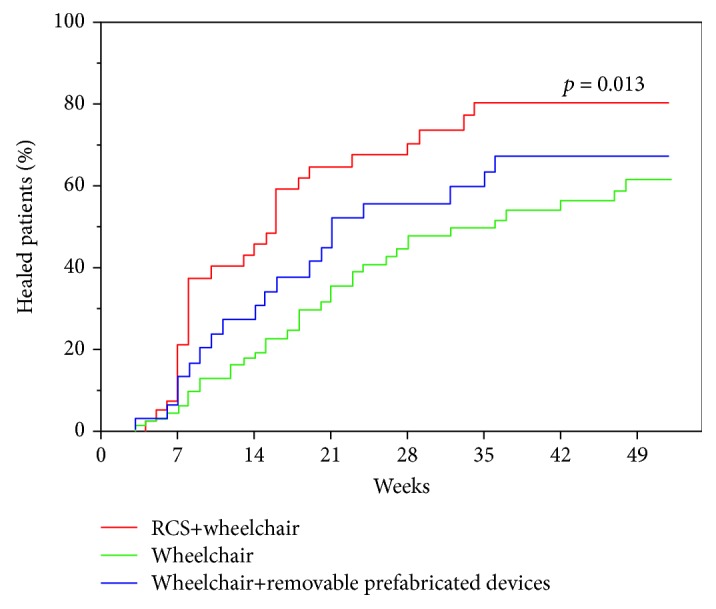
Healing of DF after surgical procedures using different off-loading devices.

Higher percentages of patients in group R (78.4% and 55.7% and 65.5%, *p* = 0.0681; Kaplan-Meier (see Figure 3); *p* = 0.013) healed for a considerably shorter time compared to the W and WP groups (13.7 vs. 20.3 and 16.5 weeks, *p* = 0.055; Table 2). Groups R and WP had significantly shorter ATB intakes than group W (*p* = 0.0019; Table 2). With regard to secondary outcomes, we observed a significantly lower number of reamputations in patients from group R compared to groups W and WP (0.16 vs. 0.7 and 0.55 number/patient; *p* = 0.028). The incidence of major amputations was significantly higher in group W compared to groups R and WP (8.2% vs. 0% and 0%; *p* = 0.023). Patients from group R were less frequently rehospitalized to a significant degree (*p* = 0.0085; Table 3).

Our subanalysis of the relation between surgery region and off-loading showed that the percentage of healed DF patients after forefoot surgical procedures was higher (but not significantly) in patients treated by an RCS+wheelchair (82.4% in group R vs. 68.9% in groups W and WP; NS). These patients healed faster (13.6 vs. 18.7; *p* = 0.064) and were treated by ATBs for a significantly shorter time (10.4 vs. 16 weeks; *p* = 0.038) compared to individuals from groups W and WP. During postoperative care, patients from group R underwent less reamputations (*p* = 0.0004), major amputations (*p* = 0.024), and rehospitalizations (*p* = 0.027) compared to the other study groups (Table 4).

There was a trend toward better healing in midfoot surgery patients provided with RCS+wheelchair compared to other study groups (73.3% vs. 45.5% of healed patients; *p* = 0.09). Wound healing was significantly faster in group R compared to groups W and WP (10 vs. 19.9 weeks; *p* = 0.005), a trend associated with shorter ATB usage in the same study group (9.7 vs. 16 weeks, *p* = 0.0043). Postoperation complications did not differ significantly between study groups. These data are further illustrated in Table 4.

We also observed a significantly higher percentage of healed patients (80% vs. 14.3%; *p* = 0.029) and a trend toward a lower number of reamputations (0%) and rehospitalizations (0.2 per patient) in patients after hindfoot surgical procedures provided with an RCS+wheelchair compared to other study groups (Table 4). Given the low number of subjects with hindfoot surgical procedures, evolutive data are inconclusive for these patients.

Based on our analysis of the possible impacts of RCS on surgical wound healing in DF patients, we found that RCS significantly increased the healing rate 2.5-fold (odds ratio (OR) 2.53, lower 95% 1.04–upper 95% 6.15, *p* = 0.037) and reduced the need for reamputations (OR 0.319, lower 95% 0.121–upper 95% 0.843, *p* = 0.0175) and rehospitalizations (OR 0.221, lower 95% 0.084–upper 95% 0.582; *p* = 0.0013) compared to patients treated by wheelchair only or a wheelchair+another removable device.

## 4. Discussion

One of the key components of DF treatment is the provision of an adequate form of lower limb off-loading [1]. The types of off-loading used for diabetic ulcers, pathological fractures, and Charcot osteoarthropathy are usually determined based on clinical findings, foot diameter, patient mobility, and comorbidities (including poor cardiovascular status and the presence of foot deformities) [16]. The gold standard for off-loading is the application of TCCs [2, 9, 17, 18]. Their effectiveness has been demonstrated in several studies, e.g., by Armstrong et al. [9], Katz et al. [19], Lavery et al. [20], and Pua et al. [21]. From a technical point of view, TCCs are considered both removable and nonremovable devices. Removable devices are particularly indicated in the case of diabetic patients not only because they are easy to handle by both patients and medical staff but also because they are readily available. However, according to a number of studies, they are not as effective as nonremovable devices [19, 22, 23] probably due to lower patient compliance with off-loading and/or a tendency toward higher physical activity [24]. As described in Morona et al.'s systematic review [24], nonremovable off-loading devices are more effective at promoting the healing of diabetic foot ulcers (hazard ratio: 1.43; [24]). A recent overview of 13 randomized controlled trials revealed that TCC and irremovable cast walkers are superior to removable cast walkers in the treatment of neuropathic, noninfected foot ulcers in patients with diabetes but without severe PAD [25]. Nevertheless, the vast majority of studies indicate that the healing time for TCC treatment is shorter than other off-loading methods [26].

So far, there are insufficient data to provide a comprehensive overview of the most effective off-loading method in DF patients following surgical procedures [23]. Not only do off-loading studies usually exclude patients with PAD, infection, and surgical procedures, they also disregard pathology locations [9]. The only papers to have accounted for the location of foot lesions are studies by Lavery et al. and Bus et al. [20, 27]. The Lavery trial included patients with forefoot ulcers, but without significant PAD. The study reported the higher effectiveness of TCC in the healing of DF ulcers (compared to other methods), an increased number of healed patients and shorter healing times [20]. Another randomized, controlled multicentre study by Bus et al. comprising 60 patients with forefoot defects reported that TCCs and other off-loading devices have the same effect on the healing of DF [27]. The above studies selectively excluded surgically treated patients and individuals with DF ulcers in the mid- or hindfoot, where based on our experience, healing is often altered.

For our DF patients under postoperative care, we mostly employed empirical data for off-loading indications. There are currently no valid studies on off-loading in specific relation to the locations of surgical procedures in DF patients. One randomized study, ORTHODIAB, is planned to redress this unmet need. This trial will try to identify the impact of using a new removable device on the healing of patients with diabetic ulceration and amputation in comparison with other prefabricated devices. The advantages of this study are that it uses orthosis to evaluate real-time off-loading while also estimating patient adherence [28]. Its limitations, in our opinion, pertain to the inclusion of a wider cross section of patients with chronic/acute amputation/resection wounds and patients with forefoot problems only [28].

The goal with our postsurgical DF patients was to heal as many wounds as possible in the shortest time. Therefore, we needed to know which type of off-loading would be the most effective with respect to the location of the surgical procedure. In our patients, amputation procedures in the area of the forefoot are usually performed in cases of osteomyelitis, nonhealing ulcers, and chronic fistulae. Midfoot or hindfoot procedures are indicated for osteomyelitis of the tarsal or calcaneal bones, nonhealing ulcers, ulcers in the region of Charcot osteoarthropathy deformities, pseudocysts, chronic fistulae, etc. These surgical procedures not only involve less extensive operations such as metatarsal osteotomies and capsulotomies [29] but also more extensive ray or transmetatarsal amputations, bone resections, and calcanectomies [30]. Our study is intended to determine the most effective off-loading method used to heal surgical procedures for various locations in DF patients and minimise the number of postoperative complications. We did not indicate nonremovable devices because the status of surgical wounds needs to be checked regularly by the medical staff and/or the patient while under postoperative care. When local findings worsen, an immediate change in therapy is recommended.

The data from the present study suggest that the most effective off-loading for DF patients with surgical procedures is the combination of a wheelchair plus an RCS. These patients achieve the highest percentages of DF healing compared to patients treated by a wheelchair alone or a combination of a wheelchair plus a removable device (almost up to 10-20%). The healing of surgical wounds such as usage of ATB therapy was significantly shortened by this off-loading method (both up to 7 weeks). This consequently leads to a reduction in the costs of DF therapy, while improving patient comfort and reducing ATB side effects experienced by patients (e.g., less allergic reactions, clostridial infections) [31] and benefitting bacterial epidemiology (lower induction of bacterial resistance) [31, 32].

Concerning the secondary endpoints of DF therapy, we observed the lowest postoperative complications in patients treated by a combination of a wheelchair plus an RCS (in group R). This group of patients underwent less frequent reamputations and rehospitalizations (a reduction of nearly 77% and 66%, respectively). There are different data on the prevalence of DF reamputations. According to Borkosky and Roukis, it is approximately 20% [33], while Thorud et al. report postoperative reamputations in up to 27% of cases [30]. In our study, we found lower prevalence in individuals treated by an RCS in group R (16.2%) compared to the W (37.7%) and WP (37.9%) groups, which probably was reflected in the reduction in hospital costs. We observed better primary and secondary outcomes of DF therapy when using a wheelchair plus RCS (in patient from group R), particularly in terms of fixing and stabilising the foot in the desired position as well as foot immobilisation. The effect of RCS is probably also connected with better patient compliance. As confirmed in a study by Waaijman et al., patients off-loaded by a wheelchair alone or a combination of a wheelchair and another removable device are less likely to continue to use the device, especially when at home [34].

The aim of our subanalysis was to assess whether the location of the surgical procedure would play a role in selecting the off-loading method and in the subsequent DF healing process. To verify the effectiveness of RCS, we have pooled the W and WP groups during the subanalysis due to small numbers of included patients. Our subanalysis revealed that in patients after forefoot procedures the number of healed patients did not differ between groups. There was a trend toward the better effect of RCS, with healing time borderline significant. However, we observed considerably less reamputations in the group of patients provided a wheelchair plus an RCS (group R). These patients were treated for shorter periods by antibiotics, thus likely minimising the risk of antibiotic resistance to causative infectious bacterial strains [35]. Also, during postoperative follow-up, these patients were less frequently rehospitalized and underwent fewer reamputations and even fewer major amputations than the cohort as a whole.

With regard to midfoot surgery, there was a trend for surgical wounds to heal better and over a significantly shorter period (reducing by almost 10 weeks) in the group provided a wheelchair and an RCS (group R) compared to other study cohorts. Thus, in this group, ATB therapy lasted for a shorter time (more than 6 weeks) and the cost of hospitalizations reduced. As part of the study, a minority of patients underwent hindfoot operations, and while the results were not statistically demonstrative, we observed a significantly higher percentage of healed patients (up to 80%) in the individuals treated by an RCS compared to other study groups (14.3%). As our data on the healing of surgical wounds in the area of the fore-, mid-, and hindfoot using various types of off-loading techniques are unique, they have yet to be compared with other studies.

Our study has several limitations. It is not a randomized controlled trial because of the relatively heterogeneous set of patients enrolled and the necessity of adopting an individual approach to off-loading according to each patient's needs and clinical findings. All these limitations could bias the results of this study. Patients were not consistently monitored for compliance and activity, as was the case in a study by Crews et al. [36], who monitored the adherence of patients treated for diabetic ulceration using removable devices. That particular study monitored patients for a relatively short time, on average, 35 days using activity monitors [36]. In our study, our ability to monitor patient activity was limited for several reasons: patients were not checked as frequently, they often came from distant parts of the country, and they were followed up for a relatively long time in our outpatient foot clinic. Other than questionnaires (compliance was determined based on patients wearing the device ≥ 50% during daytime), we had no monitoring systems at our disposal [34, 36]. However, in our experience, postoperative patient adherence to off-loading is higher than in patients with chronic wounds.

Other limitation is the inclusion of different numbers of patients to study groups that was given by inclusion criteria. We have treated surgically a wide spectrum of patients with diabetic foot; however, off-loading methods were frequently changed during the postsurgical follow-up. Since we aimed to clearly show how effective certain off-loading devices are, we rather included only a part of well-defined subjects into our study. The distribution of patients in study groups was therefore not equivalent. Nevertheless, based on power analysis for healing, this study have sufficient strength. Moreover, study groups differed in age and the incidence of PAD as well as inpatient/outpatient rates. Neither age nor PAD influenced significantly based on statistical analysis the healing of study subjects during the follow-up period. That is maybe because of relatively high mean TcPO_2_ values in all study groups that varied from 38 to 43 mmHg. Another inpatient/outpatient rates in study groups have been given due to the enrollment of hospitalized patients only into group R; other groups included mostly hospitalized patients with a small proportion of outpatient subjects. This is due to better timing for splint making, since we always apply the splint every 2nd or 3rd day after the patient's surgery. Sometimes it can be hard to fulfill this schedule in an outpatient setting.

In conclusion, our study provides a comprehensive report on the types of off-loading that might be indicated in DF patients following surgical procedures. The data suggest that a customized removable splint may be superior as it increases the likelihood of DF healing 2.5-fold, reduces the risk of reamputations by almost threefold, and lessens the risk of rehospitalizations by almost fivefold. RCSs are further able to reduce healing time and the duration of ATB treatment. We contend that when forefoot surgery patients are treated by an RCS, fewer postoperative complications are likely to occur. In the case of surgical operations in the mid- and hindfoot, a wheelchair plus an RCS may lead to better DF healing. We look forward to further studies that may confirm, modify, or refute these findings. A randomized study should confirm the interest of individualized removable casting techniques.

## Figures and Tables

**Table 1:** A comparison of basic characteristics among the study groups.

Evaluated parameters	Group R (*n* = 37)	Group W (*n* = 61)	Group WP (*n* = 29)	*p* value
Age (years)	57.9 ± 10.7^∗^	64.6 ± 12.2	65.6 ± 10.1	*p* = 0.007
Diabetes duration (years)	20.3 ± 11.2	21.7 ± 12.5	22.3 ± 13.6	NS
HbA1c (%/mmol/mol)	7.9 ± 3.8/62.9 ± 17.7	7.9 ± 4.1/62.3 ± 20.9	7.6 ± 3.5/59.3 ± 15.2	NS
BMI (kg·m^−2^)	30.1 ± 5.5	30.7 ± 5.5	29.2 ± 5.5	NS
Haemodialysis (% of patients)	8.1	11.5	17.2	NS
Organ transplantation (% of patients)	16.2	8.2	6.9	NS
Peripheral arterial disease (% of patients)	51	78.7^†^	69	*p* = 0.019
TcPO_2_ (mmHg)	43.1 ± 12.9	37.5 ± 19.2	41.4 ± 14.7	NS
Osteomyelitis (% of patients)	70.3	62.3	82.8	NS
Outpatient/inpatient procedures (%/% of patients)	0/100^‡^	13.1/86.9	34.5/65.5	*p* < 0.0001
Revascularisation during follow-up (% of patients)	13.5	31.2	27.6	NS

Data are presented as means ± SD; HbA1c: glycated haemoglobin values according to DCCT/IFCC; BMI: body mass index; TcPO_2_: transcutaneous oxygen pressure; NS: nonsignificant; *p*: value of significance compared among the study groups (group R: patients treated by a combination of a wheelchair plus a removable contact splint, group W: wheelchair only, and group WP: wheelchair plus a removable device) detected by one-way ANOVA, comparisons of all pairs based on Turkey-Kramer analysis, contingency analysis, and the Cochran-Armitage trend test; ^∗^group R vs. other study groups; ^†^group W compared to group R; ^‡^group R compared to group WP.

**Table 2:** A comparison of primary outcomes of DF healing after foot surgery among the study groups.

Evaluated parameters	Group R (*n* = 37)	Group W (*n* = 61)	Group WP (*n* = 29)	*p* value
Hospital stay (days)	15 ± 8	18.8 ± 9.5^∗^	14.3 ± 9.1	*p* = 0.04
Hospitalization costs (€/1 hospitalization)	3787 ± 2500	5378 ± 3303^∗^	4013 ± 2961	*p* = 0.025
Healing of DF (% of patients)	78.4^†^	55.7	65.5	*p* = 0.068
Healing time (weeks)	13.7 ± 8.5^†^	20.3 ± 11.9	16.5 ± 9.94	*p* = 0.055
Length of antibiotic therapy (weeks)	11 ± 10.3^†^	18 ± 12.6	11.9 ± 8.2	*p* < 0.002

Data are presented as means ± SD; DF: diabetic foot; NS: nonsignificant; *p*: value of significance among the study groups (group R: patients treated by a combination of a wheelchair plus a removable contact splint, group W: wheelchair only, and group WP: wheelchair plus a removable device) detected by one-way ANOVA, comparisons of all pairs based on Turkey-Kramer analysis, the Wilcoxon/Kruskal-Wallis tests; nonparametric comparisons for all pairs based on the Steel-Dwass method, contingency analysis, and the Cochran-Armitage trend test; ^∗^group W vs. group R and WP; ^†^group R compared to group W.

**Table 3:** A comparison of secondary outcomes of DF therapy after foot surgery among the study groups.

Evaluated parameters	Group R (*n* = 37)	Group W (*n* = 61)	Group WP (*n* = 29)	*p* value
Reamputation (number/1 patient)	0.16 ± 0.37^∗^	0.7 ± 1.1	0.55 ± 0.9	*p* = 0.028
Major amputation (% of patients)	0	8.2^†^	0	*p* = 0.023
Rehospitalization (number/1 patient)	0.3 ± 0.8^‡^	0.87 ± 1.2	0.79 ± 1.1	*p* = 0.0085
Rehospitalization stay (days)	15.5 ± 10.4	22.6 ± 16	19.8 ± 13.7	NS

Data are presented as means ± SD; DF: diabetic foot; NS: nonsignificant; *p*: value of significance among the study groups (group R: patients treated by a combination of a wheelchair plus a removable contact splint, group W: wheelchair only, and group WP: wheelchair plus a removable device) detected by one-way ANOVA, comparisons of all pairs based on Turkey-Kramer analysis, the Wilcoxon/Kruskal-Wallis tests; and nonparametric comparisons for all pairs based on the Steel-Dwass method; ^∗^group W compared to group R; ^†^group W compared to groups R and WP; ^‡^group R compared to groups W and WP.

**Table 4:** A comparison of primary and secondary outcomes of DF therapy among the study groups according to surgical procedure location.

	Forefoot surgery			Midfoot surgery			Hindfoot surgery		
Evaluated parameters	Group R (*n* = 17)	Group W+group WP (*n* = 61)	*p* value	Group R (*n* = 15)	Group W+group WP (*n* = 22)	*p* value	Group R (*n* = 5)	Group W+group WP (*n* = 7)	*p* value
Hospital stay (days)	14.2 ± 7.9	16.6 ± 10	NS	14.9 ± 9	19.2 ± 9.2	NS	18 ± 6	19.7 ± 5.1	NS
Hospitalization costs (€/1 hospitalization)	3754 ± 2734	4769 ± 3278	NS	3615 ± 2617^∗^	5666 ± 3618	*p* = 0.07	4551 ± 1426	6073 ± 1923	NS
Healing of DF (% of patients)	82.4	68.9	NS	73.3^∗^	45.5	*p* = 0.09	80^∗^	14.3	*p* = 0.029
Healing time (weeks)	13.6 ± 7.1^∗^	20.4 ± 13.3	*p* = 0.06	10 ± 5.76^∗^	19.9 ± 8.2	*p* = 0.005	24.5 ± 12	20 ± 0	NS
Length of antibiotic therapy (weeks)	10.4 ± 8.5^∗^	16 ± 12.4	*p* = 0.038	9.7 ± 11.4^∗^	16 ± 10	*p* = 0.0043	17.2 ± 12.7	16.4 ± 9.4	NS
Reamputation (number/1 patient)	0.18 ± 0.39^∗^	0.77 ± 1	*p* = 0.0004	0.2 ± 0.41	0.36 ± 0.8	NS	0	0.57 ± 1.4	NS
Major amputation (% of patients)	0^∗^	8.2	*p* = 0.024	0	0	NS	0	0	NS
Rehospitalization (number/1 patient)	0.35 ± 0.86^∗^	0.95 ± 1.18	*p* = 0.027	0.27 ± 0.8	0.55 ± 0.8	NS	0.2 ± 0.4	0.86 ± 1.4	NS
Rehospitalization stay (days)	16.3 ± 9.5	22.5 ± 15.5	NS	19 ± 15.6	17.1 ± 99	NS	6	26 ± 19.9	NS

Data are presented as means ± SD; DF: diabetic foot; NS: nonsignificant; *p*: value of significance among the study groups (group R: patients treated by a combination of a wheelchair plus a removable contact splint, group W: wheelchair only, and group WP: wheelchair plus a removable device) detected by one-way ANOVA. Comparisons of all pairs based on Tukey-Kramer analysis and the Wilcoxon/Kruskal-Wallis tests; nonparametric comparisons for all pairs based on the Steel-Dwass method; ^∗^group W compared to groups W and WP.

## Data Availability

The data used to support the finding of this study are available from the corresponding author upon request.

## References

[1] (2015). *The 2015 IWGDF guidance documents on prevention and management of foot problems in diabetes: development of an evidence-based global consensus*.

[2] Hingorani A., LaMuraglia G. M., Henke P. (2016). The management of diabetic foot: a clinical practice guideline by the Society for Vascular Surgery in collaboration with the American Podiatric Medical Association and the Society for Vascular Medicine. *Journal of Vascular Surgery*.

[3] Armstrong D. G., Boulton A. J. M., Bus S. A. (2017). Diabetic foot ulcers and their recurrence. *New England Journal of Medicine*.

[4] Schaper N. C., van Netten J., Apelqvist J., Lipsky B. A., Bakker K., International Working Group on the Diabetic Foot (IWGDF) (2017). Prevention and management of foot problems in diabetes: a Summary Guidance for Daily Practice 2015, based on the IWGDF guidance documents. *Diabetes Research and Clinical Practice*.

[5] Cavanagh P. R., Bus S. A. (2011). Off-loading the diabetic foot for ulcer prevention and healing. *Plastic and Reconstructive Surgery*.

[6] Elraiyah T., Prutsky G., Domecq J. P. (2016). A systematic review and meta-analysis of off-loading methods for diabetic foot ulcers. *Journal of Vascular Surgery*.

[7] Bus S. A., Armstrong D. G., van Deursen R. W. (2016). IWGDF guidance on footwear and offloading interventions to prevent and heal foot ulcers in patients with diabetes. *Diabetes/Metabolism Research and Reviews*.

[8] Healy A., Naemi R., Chockalingam N. (2014). The effectiveness of footwear and other removable off-loading devices in the treatment of diabetic foot ulcers: a systematic review. *Current Diabetes Reviews*.

[9] Armstrong D. G., Nguyen H. C., Lavery L. A., van Schie C. H. M., Boulton A. J. M., Harkless L. B. (2001). Off-loading the diabetic foot wound: a randomized clinical trial. *Diabetes Care*.

[10] Armstrong D. G., Lavery L. A., Wu S., Boulton A. J. M. (2005). Evaluation of removable and irremovable cast walkers in the healing of diabetic foot wounds: a randomized controlled trial. *Diabetes Care*.

[11] Piaggesi A., Macchiarini S., Rizzo L. (2007). An off-the-shelf instant contact casting device for the management of diabetic foot ulcers: a randomized prospective trial versus traditional fiberglass cast. *Diabetes Care*.

[12] Faglia E., Caravaggi C., Clerici G. (2010). Effectiveness of removable walker cast versus nonremovable fiberglass off-bearing cast in the healing of diabetic plantar foot ulcer: a randomized controlled trial. *Diabetes Care*.

[13] Fejfarová V., Jirkovská A., Křížová M., Skibová J. (2005). Účinnost léčby snímatelnými kontaktními fixacemi u pacientů s neuropatickými ulceracemi, akutní Charcotovou osteoarthropatií a neuropatickými frakturami. *Vnitřní Lékařství*.

[14] Jeon B. J., Choi H. J., Kang J. S., Tak M. S., Park E. S. (2017). Comparison of five systems of classification of diabetic foot ulcers and predictive factors for amputation. *International Wound Journal*.

[15] Fejfarová V., Jirkovská A., Bém R. (2016). Special contact splints in postoperative care for patients with the diabetic foot. *Rozhledy v Chirurgii*.

[16] Fejfarová V., Jirkovská A. (2015). *Léčba Syndromu Diabetické Nohy Odlehčením*.

[17] Cavanagh P. R., Bus S. A. (2010). Off-loading the diabetic foot for ulcer prevention and healing. *Journal of Vascular Surgery*.

[18] Stark C., Murray T., Gooday C. (2016). 5 year retrospective follow-up of new cases of Charcot neuroarthropathy—a single centre experience. *Foot and Ankle Surgery*.

[19] Katz I. A., Harlan A., Miranda-Palma B. (2005). A randomized trial of two irremovable off-loading devices in the management of plantar neuropathic diabetic foot ulcers. *Diabetes Care*.

[20] Lavery L. A., Higgins K. R., La Fontaine J., Zamorano R. G., Constantinides G. P., Kim P. J. (2015). Randomised clinical trial to compare total contact casts, healing sandals and a shear-reducing removable boot to heal diabetic foot ulcers. *International Wound Journal*.

[21] Pua B. B., Muhs B. E., Maldonado T., Ben-Arie E., Sheehan P., Gagne P. J. (2006). Total-contact casting as an adjunct to promote healing of pressure ulcers in amputees. *Vascular and Endovascular Surgery*.

[22] Snyder R. J., Frykberg R. G., Rogers L. C. (2014). The management of diabetic foot ulcers through optimal off-loading: building consensus guidelines and practical recommendations to improve outcomes. *Journal of the American Podiatric Medical Association*.

[23] Bus S. A., van Deursen R. W., Armstrong D. G. (2016). Footwear and offloading interventions to prevent and heal foot ulcers and reduce plantar pressure in patients with diabetes: a systematic review. *Diabetes/Metabolism Research and Reviews*.

[24] Morona J. K., Buckley E. S., Jones S., Reddin E. A., Merlin T. L. (2013). Comparison of the clinical effectiveness of different off-loading devices for the treatment of neuropathic foot ulcers in patients with diabetes: a systematic review and meta-analysis. *Diabetes/Metabolism Research and Reviews*.

[25] Health Quality Ontario (2017). Fibreglass total contact casting, removable cast walkers, and irremovable cast walkers to treat diabetic neuropathic foot ulcers: a health technology assessment. *Ontario Health Technology Assessment Series*.

[26] Van De Weg F. B., Van Der Windt D. A. W. M., Vahl A. C. (2008). Wound healing: total contact cast vs. custom-made temporary footwear for patients with diabetic foot ulceration. *Prosthetics and Orthotics International*.

[27] Bus S. A., van Netten J. J., Kottink A. I. R. (2018). The efficacy of removable devices to offload and heal neuropathic plantar forefoot ulcers in people with diabetes: a single-blinded multicentre randomised controlled trial. *International Wound Journal*.

[28] Mohammedi K., Potier L., François M. (2016). The evaluation of off-loading using a new removable oRTHOsis in DIABetic foot (ORTHODIAB) randomized controlled trial: study design and rational. *Journal of Foot and Ankle Research*.

[29] Botezatu I., Laptoiu D. (2016). Minimally invasive surgery of diabetic foot – review of current techniques. *Journal of Medicine and Life*.

[30] Thorud J. C., Jupiter D. C., Lorenzana J., Nguyen T. T., Shibuya N. (2016). Reoperation and reamputation after transmetatarsal amputation: a systematic review and meta-analysis. *The Journal of Foot and Ankle Surgery*.

[31] Game F. (2010). Management of osteomyelitis of the foot in diabetes mellitus. *Nature Reviews Endocrinology*.

[32] Lipsky B. A. (2016). Diabetic foot infections: current treatment and delaying the ‘post-antibiotic era’. *Diabetes/Metabolism Research and Reviews*.

[33] Borkosky S. L., Roukis T. S. (2012). Incidence of re-amputation following partial first ray amputation associated with diabetes mellitus and peripheral sensory neuropathy: a systematic review. *Diabetic Foot & Ankle*.

[34] Waaijman R., Keukenkamp R., de Haart M., Polomski W. P., Nollet F., Bus S. A. (2013). Adherence to wearing prescription custom-made footwear in patients with diabetes at high risk for plantar foot ulceration. *Diabetes Care*.

[35] Fejfarová V., Jirkovská A., Petkov V., Bouček P., Skibová J. (2004). Comparison of microbial findings and resistance to antibiotics between transplant patients, patients on hemodialysis, and other patients with the diabetic foot. *Journal of Diabetes and its Complications*.

[36] Crews R. T., Shen B. J., Campbell L. (2016). Role and determinants of adherence to off-loading in diabetic foot ulcer healing: a prospective investigation. *Diabetes Care*.

